# Disaster Vulnerability in Long-Term Care: The Importance of Social and Organizational Connections

**DOI:** 10.1093/geroni/igab046.774

**Published:** 2021-12-17

**Authors:** Lindsay Peterson, Debra Dobbs, Joseph June, David Dosa, Kathryn Hyer

**Affiliations:** 1 University of South Florida, Bradenton, Florida, United States; 2 University of South Florida, School of Aging Studies, University of South Florida, Florida, United States; 3 University of South Florida, Tampa, Florida, United States; 4 Brown University, Providence, Rhode Island, United States; 5 University of South Florida, University of South Florida, Florida, United States

## Abstract

The risks to older adults in nursing homes (NHs) and assisted living communities (ALCs) exposed to disasters are evident in prior research. However, little research has been conducted to understand the factors related to facilities’ vulnerability. This research examined NH and ALC experiences during Hurricane Irma in 2017. Qualitative interviews were conducted with representatives of facilities (N=100), transcripts were analyzed using Atlas.ti version 8. Team members met to reach consensus on codes and major themes and subthemes, which they analyzed using a conceptual model designed to identify factors related to the disaster vulnerability in long-term care (LTC). We found physical factors (e.g. location, physical characteristics) are important, but physical strength is not enough. Multiple social/organizational factors are critical. Results indicate managing a major disaster and protecting LTC residents involve social and organizational connections across a range of groups from staff and family members to emergency mangers and neighborhood associations.

